# Biomechanical interactions of *Schistosoma mansoni* eggs with vascular endothelial cells facilitate egg extravasation

**DOI:** 10.1371/journal.ppat.1010309

**Published:** 2022-03-22

**Authors:** Yi-Ting Yeh, Danielle E. Skinner, Ernesto Criado-Hidalgo, Natalie Shee Chen, Antoni Garcia-De Herreros, Nelly El-Sakkary, Lawrence Liu, Shun Zhang, Adithan Kandasamy, Shu Chien, Juan C. Lasheras, Juan C. del Álamo, Conor R. Caffrey

**Affiliations:** 1 Department of Mechanical and Aerospace Engineering, University of California, San Diego, La Jolla, California, United States of America; 2 Department of Bioengineering, University of California, San Diego, La Jolla, California, United States of America; 3 Institute of Engineering in Medicine, University of California, San Diego, La Jolla, California, United States of America; 4 Center for Discovery and Innovation in Parasitic Diseases (CDIPD), Skaggs School of Pharmacy and Pharmaceutical Sciences, University of California, San Diego, La Jolla, California, United States of America; 5 Department of Mechanical Engineering, University of Washington, Seattle, Washington, United States of America; 6 Center for Cardiovascular Biology, University of Washington, Seattle Washington, United States of America; 7 Institute for Stem Cell and Regenerative Medicine, University of Washington, Seattle Washington, United States of America; George Washington University, UNITED STATES

## Abstract

The eggs of the parasitic blood fluke, *Schistosoma*, are the main drivers of the chronic pathologies associated with schistosomiasis, a disease of poverty afflicting approximately 220 million people worldwide. Eggs laid by *Schistosoma mansoni* in the bloodstream of the host are encapsulated by vascular endothelial cells (VECs), the first step in the migration of the egg from the blood stream into the lumen of the gut and eventual exit from the body. The biomechanics associated with encapsulation and extravasation of the egg are poorly understood. We demonstrate that *S*. *mansoni* eggs induce VECs to form two types of membrane extensions during encapsulation; filopodia that probe eggshell surfaces and intercellular nanotubes that presumably facilitate VEC communication. Encapsulation efficiency, the number of filopodia and intercellular nanotubes, and the length of these structures depend on the egg’s vitality and, to a lesser degree, its maturation state. During encapsulation, live eggs induce VEC contractility and membranous structures formation in a Rho/ROCK pathway-dependent manner. Using elastic hydrogels embedded with fluorescent microbeads as substrates to culture VECs, live eggs induce VECs to exert significantly greater contractile forces during encapsulation than dead eggs, which leads to 3D deformations on both the VEC monolayer and the flexible substrate underneath. These significant mechanical deformations cause the VEC monolayer tension to fluctuate with the eventual rupture of VEC junctions, thus facilitating egg transit out of the blood vessel. Overall, our data on the mechanical interplay between host VECs and the schistosome egg improve our understanding of how this parasite manipulates its immediate environment to maintain disease transmission.

## Author summary

Schistosomiasis, which infects over 200 million people, is a painful disease of poverty that is caused by inflammatory responses to the *Schistosoma* blood fluke’s eggs. To continue the parasite’s life cycle, eggs must escape the blood vessels and migrate through tissues of the host to the alimentary canal for exit into the environment. The biomechanical processes that help the immobile eggs to cross the blood vessel’s vascular endothelial cells (VECs) as the first step in this migration are not understood. We found that live but not dead eggs induce VECs to crawl over and encapsulate them. VECs in contact with live eggs make membranous extensions (filopodia) to explore the egg’s surface and also form long intercellular nanotubes to communicate with neighboring cells. VECs stimulate particular (Rho/ROCK) biochemical pathways to increase cell contractility and the forces generated are large enough to eventually break the junctions between cells and allow passage of the eggs into the underlying tissue. Our findings show how schistosome eggs activate and interact with VECs to initiate their escape from the bloodstream.

## Introduction

Schistosomiasis is caused by several species of the *Schistosoma* blood fluke. Transmitted by freshwater snails, the disease is prevalent in sub-Saharan Africa and parts of Southeast Asia and South America, with approximately 220 million people infected [1]. During infection with *Schistosoma mansoni*, paired male and female worms wander through the mesenteric and hepatic portal venous systems that drain the alimentary canal. Females lay hundreds of eggs per day, and these come into direct contact with vascular endothelial cells (VECs) [2–4]. VECs first migrate over and encapsulate the eggs, initiating an inflammatory granulomatous response that facilitates egg extravasation. Egg extravasation is critical for subsequent granuloma-mediated egg transport through the gut wall into the lumen, and eventually, the release of eggs into the external environment with the feces [3,5–8]. Eggs that fail to extravasate are swept away by the blood flow and become trapped in the liver, where they induce granulomata and eventually fibrosis which results in pain and malaise with often life-threatening sequela over the course of years or even decades [9–11].

Extravasation of the schistosome egg requires mechanical forces to push the egg toward the extravascular space and bring it into direct contact with the blood vessel’s basement membrane. However, the rigidity of the eggshell limits the transmission of appreciable forces between the egg and its surroundings [3,4,12]. Muscular contractions of the female worm and/or migration of the host’s VECs over the egg could generate the forces necessary for egg transmigration [13]. Although the movement forces of the male worm have been quantified [14], there are no measurements of female-generated worm forces, including those during egg deposition. VEC actomyosin contractility, coupled with substrate adhesion, create tensile forces that are transmitted between neighboring cells via adherens junctions, and, together, these contribute to the generation of mechanical tension at the tissue level [15]. Tensional homeostasis within cell monolayers plays a role in a wide range of processes such as shear stress mechanosensing [16], leukocyte trafficking [17], and host-pathogen interactions [18,19]. However, the VEC response to schistosome egg contact, and specifically whether and how VECs remodel their actomyosin cytoskeleton and actively generate mechanical forces to drive the encapsulation of eggs, are poorly understood.

Here, we first analyzed the ultrastructure of *S*. *mansoni* eggs and VECs during the encapsulation process and identified two types of VEC membrane extensions: filopodia and intercellular nanotubes (NTs). We showed that the number and length of filopodia and NTs during encapsulation of eggs are influenced by the age and vitality of the parasite embryo. We also performed three-dimensional traction force microscopy (3D-TFM) measurements on cultured VEC monolayers to show that live but not dead eggs increased VEC contractility, which resulted in eggs being pushed against the substrate to eventually rupture the VEC intercellular junctions. Together, our data demonstrate a series of intricate and coordinated biomechanical events that facilitate egg extravasation into the underlying host tissues.

## Results

### VECs form actin-rich filopodia structures that physically probe eggshell microspines during encapsulation of *S*. *mansoni* eggs

**Fig 1:**
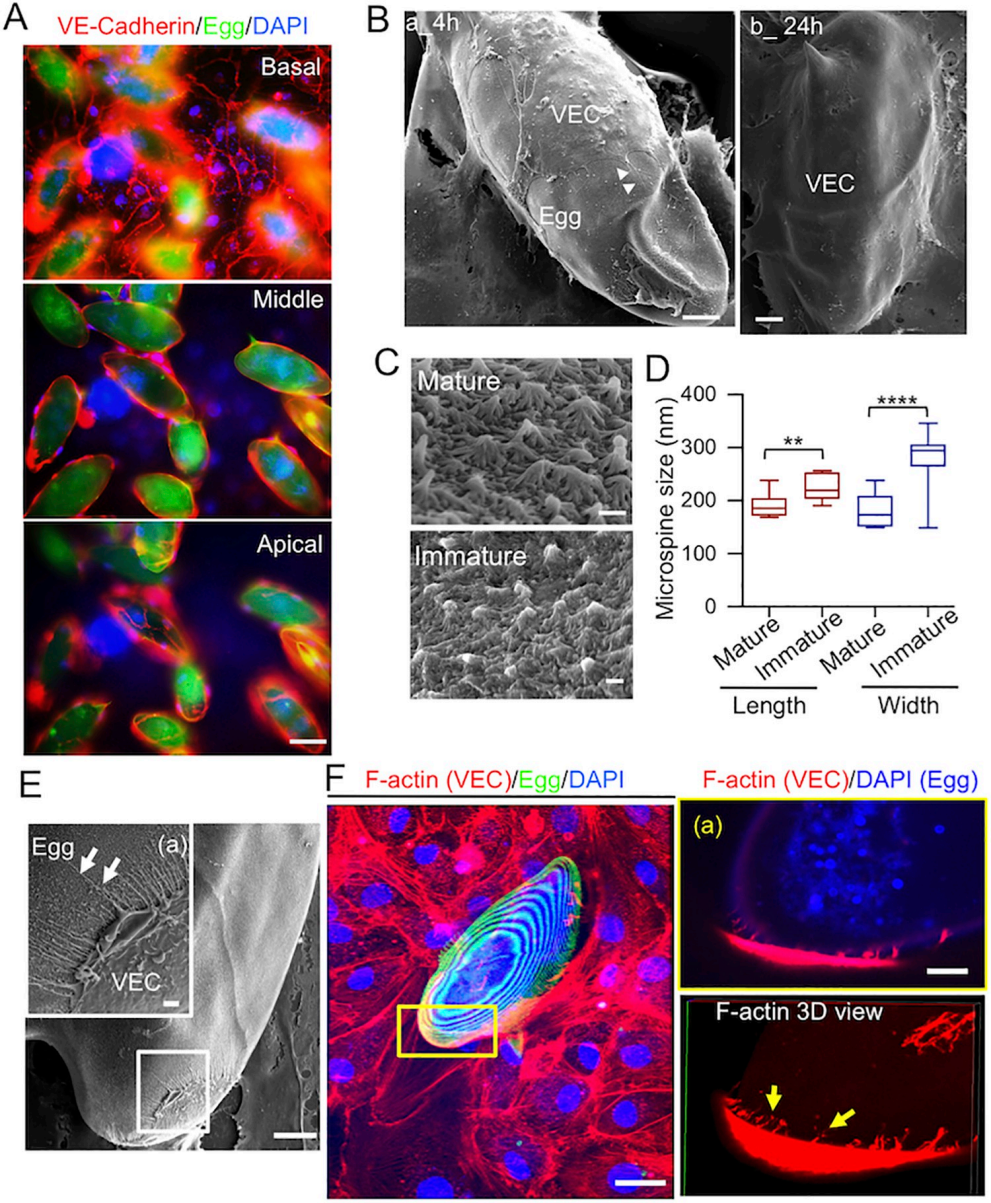
VECs generate filopodia while encapsulating *S*. *mansoni* eggs. **(A)** Immunostaining shows VECs (VE-Cadherin, red) on the basal, middle and apical areas of live *S*. *mansoni* mature eggs, which are auto-fluorescently green. DAPI was used to stain for cell nuclei. Scale bar: 50 μm. **(B)** SEM images demonstrating the interaction between VECs and the eggshell: (a) At 4 h, part of an eggshell was covered by VECs (scale bar: 10 μm), Arrowheads point VEC membrane protrusions on the eggshell; (b*)* at 24 h, the eggshell was fully covered by VECs (scale bar: 10 μm). **(C)** Image of microspines in mature and immature eggshells (scale bar: 0.2 μm). **(D)** Quantification of the length and width of microspines in mature and immature eggshells. ** and **** indicate respectively p < 0.01 and p < 0.0001 by Student’s *t-*test; 12–15 microspines were quantified in each condition. **(E)** SEM image of the interaction between the *S*. *mansoni* egg and VEC filopodia after 4 h (scale bar = 10 μm); (a) enlarged image of the boxed region of interest showing VEC filopodia (arrows) on the eggshell surface. Scale bar: 1 μm. **(F)** Confocal microscopy images of actin filaments. VECs and eggs were fixed, and immunostained with rhodamine phalloidin (red). Scale bar: 20 μm. (a) Enlarged image of the boxed region of interest (showing the presence of VEC actin-rich filopodia during VEC encapsulation of the egg. Scale bar: 5 μm. Lower panel is the 3D view and arrows indicate actin filaments on the eggshell surface.

To investigate how VECs encapsulate *S*. *mansoni* eggs, we placed mature eggs onto VEC monolayers cultured *in vitro*. After 24 h, the VECs had fully encapsulated the eggs as evidenced by immunostaining of the endothelial junction protein VE-cadherin, which was detected in all z-planes of view (basal, middle and apical; Fig 1A). We also imaged the interactions between mature eggs and VECs using scanning electron microscopy (SEM) at 4 and 24 h (Fig 1B). At 4 h, VECs protruded membrane extensions called filopodia to physically interact with the eggs; by 24 h, the VECs had completely covered the eggs. Following their release by female worms, immature eggs interact with VECs and become mature over the course of several days [20–22]. Immature eggs are about two-thirds the length and width of mature eggs (S1 Table). DAPI staining showed that most of the nuclei in immature eggs were evenly dispersed throughout the embryo, whereas those in more mature eggs have generated a well-defined circular neural mass in the center of the developing miracidium (S1B Fig and S1 Table). Furthermore, SEM showed that the surface of the eggshell was covered by microspine structures (Fig 1C), as previously reported [23]. By comparing mature and immature eggs, we found that similar microspines were present on both with an average length and width of ~200 nm (Fig 1C and 1D), although the microspine shapes on immature eggs were more irregular (Fig 1C). VECs developed long filopodia that intimately contacted the egg microspines (Fig 1E). Using confocal microscopy and phalloidin immunostaining, we confirmed the presence of F-actin in these VEC filopodia (Fig 1F). In particular, F-actin-rich filopodia were observed on the eggshell’s apical end (Fig 1F). The filopodia were integrated into the F-actin cytoskeleton of the VECs, as evidenced by tracking these structures in subsequent z-planes (S2 Fig and S1 Movie).

### In addition to filopodia, VECs generate intercellular nanotubes during the encapsulation of *S*. *mansoni* eggs

**Fig 2:**
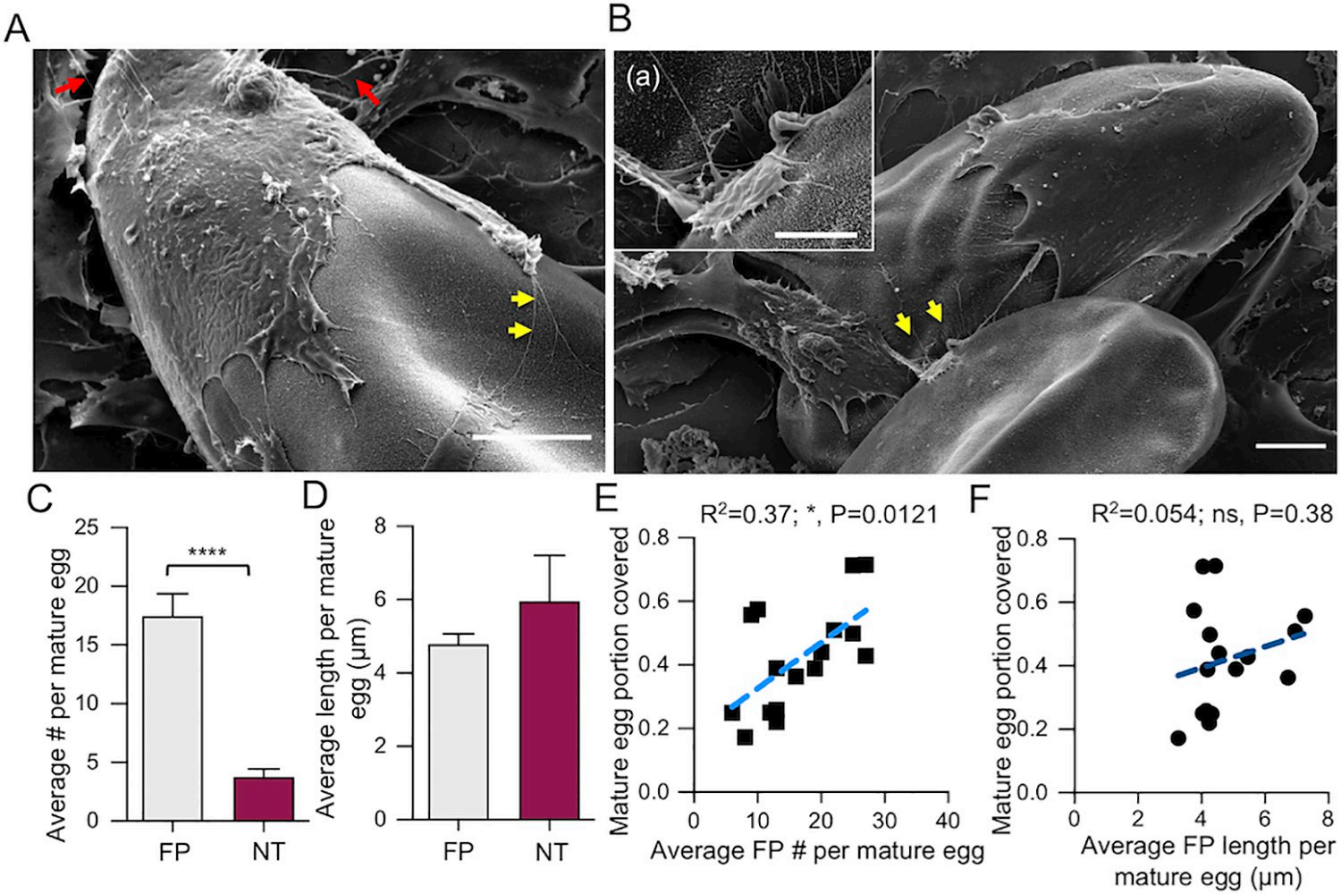
VECs interacting with *S*. *mansoni* eggs display two types of membrane protrusions. **(A)** During encapsulation, VECs extend filopodia (FP; yellow arrows), to probe the egg surface, and intercellular nanotubes (NTs; red arrows) to connect with neighboring cells (scale bar: 10 μm). **(B)** Representative SEM image to illustrate how filopodial probing facilitates VEC migration over eggs: (a) enlarged image of the probing FP corresponding to the region indicated by the yellow arrowheads (scale bar: 4 μm). **(C)** Average number of FPs and NTs formed per mature egg after 4 h. **(D)** Average length of FPs and NTs formed per mature egg after 4 h. Bar plots in **C** and **D** represent mean ± s.e.m. **** indicate p < 0.0001 by Student’s *t*-test. **(E)** Egg portion covered by VECs as a function of average FP number. **(F)** Egg portion covered by VECs as a function of average FP length. For both **E** and **F**, * indicates statistically significant Pearson correlation (p = 0.0121). For **C—F**, a total of 16 mature eggs from three independent experiments were analyzed.

After 4 h of co-culturing mature eggs with VECs, SEM revealed the presence of VEC intercellular nanotubes (NTs) in addition to filopodia on *S*. *mansoni* eggs. NTs are specialized membrane extensions that connect neighboring cells [24,25] and mediate cell-cell communications by internal transport of signaling molecules such as proteins, lipids and nucleic acids [26]. During encapsulation of the egg, VECs were observed to elaborate NTs from their trailing edges which maintained connections with the basal endothelium (Fig 2A). At the same time, filopodia were observed at the advancing front of the VECs exploring the egg surface (Fig 2A and 2B).

The average numbers of VEC filopodia and intercellular NTs per egg after a 4 h interaction were approximately 17 and 4, respectively (being significantly different from each other at p < 0.0001). However, although the average length of filopodia and NTs per egg was not significantly different (p > 0.05), being 4.78 ± 0.28 μm and 5.96 ± 1.24 μm, respectively (Fig 2C and 2D). Next, we analyzed whether VEC filopodia number and length correlated with the portion of the egg covered by VECs. There was a modest correlation for filopodia number (R^2^ = 0.37, p = 0.01, Fig 2E), but there was no correlation for filopodia length (R^2^ = 0.054, p = 0.38; Fig 2F). Together, these data demonstrate that there are two distinct types of VEC membrane protrusions, *i*.*e*., filopodia and NTs, during the egg encapsulation process, and that there is a positive correlation between VEC filopodia number and the cells’ ability to encapsulate eggs. The data may suggest that during the egg-VEC interaction, VECs favor exploring their environment over communicating with neighboring cells by generating more forward-projecting filopodia than rear-facing intercellular NTs.

### Live eggs constitute a greater stimulus than dead eggs to the encapsulation process by VECs

**Fig 3:**
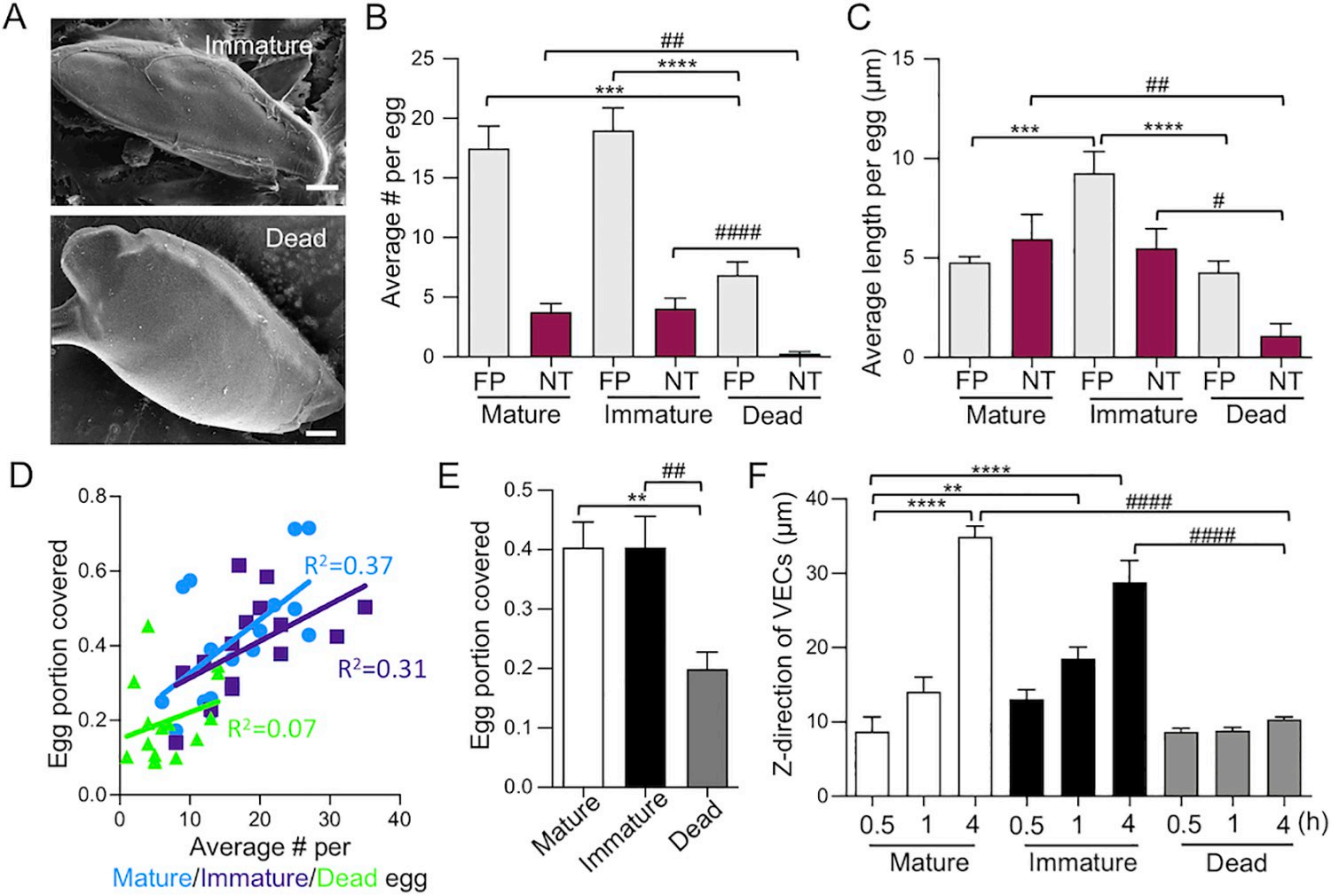
Encapsulation of *S*. *mansoni* eggs by VEC filopodia depends on egg vitality. **(A)** SEM images of VECs encapsulating a live immature egg and a dead mature egg after 4 h (scale bar: 10 μm). **(B)** Number of FPs and NTs per egg (mean ± s.e.m.). *** and **** indicate p < 0.001 and p < 0.0001 when comparing FPs. ## and #### indicate p < 0.01 and p < 0.0001 when comparing NTs. **(C)** Length of FP and NTs per egg (mean ± s.e.m.). *** and **** indicate p < 0.001 and p < 0.0001 when comparing FPs. # and ## indicate p < 0.05 and p<0.01 when comparing NTs. For **B** and **C**, a one-way ANOVA with Tukey’s post-test was used. **(D)** The portion of eggs covered by VECs as a function of the average number of FPs per egg at 4 h. Mature (light blue circles) and immature live eggs (purple squares) show significant Pearson correlations which p value = 0.012 and 0.029, respectively), whereas dead eggs do not (p = 0.075). **(E)** Portion of live mature (white), immature (black) and dead (gray) eggs covered by VECs after 4 h. ** indicates p < 0.01 and ## indicates p < 0.01 using a one-way ANOVA with Tukey’s post-test. For **A**–**E**, a total of 16, 15 and 14 live mature, live immature and dead eggs from three independent experiments were analyzed. **(F)** Quantification of the encapsulation dynamics as a function of the maximum height (z-direction; mean ± s.e.m.) reached by VECs at different time points. ** and **** indicate p < 0.01 and p < 0.0001, respectively, using a one-way ANOVA with Dunnett’s post-test. #### indicates p < 0.0001 when comparing 4 h mature, immature and dead eggs, using a one-way ANOVA with Tukey’s post-test. Across three independent experiments, 7–12 eggs were analyzed for each condition.

To understand whether the induction of VEC filopodia was influenced by the embryo’s developmental state and vitality, we tested the response of VECs to live immature and mature eggs, as well as mature eggs that had been killed with sodium azide (hereafter referred to as dead eggs). The vitality of the live mature eggs was confirmed by their ability to hatch in water under a bright light for 40 min. Hatching efficiency was ~80% (S1A Fig); also, we observed that those eggs capable of hatching always contained a moving miracidium (S2 Movie). SEM imaging showed that the average number of filopodia and NTs per egg depended on egg vitality (~2.5-fold more in live eggs, p = 0.0002, mature *vs*. dead; p < 0.0001, immature *vs*. dead) but not on the developmental state after a 4 h incubation with VECs (Fig 3A and 3B). Immature eggs induced VECs to form significantly (~2-fold) longer filopodia than either mature (p = 0.0002) or dead eggs (p < 0.0001). For NTs, length was a function of egg vitality (~5-fold longer in both mature (p = 0.0041) and immature eggs (p = 0.01), *vs*. dead eggs; Fig 3C) but not development.

Next, we investigated whether, after 4 h, there was a correlation between the number of VEC filopodia per egg and the portion of the egg covered by VECs, for each of the three egg preparations (Fig 3D). There was a modest correlation for live mature (R^2^ = 0.37, p = 0.012) and immature eggs (R^2^ = 0.31, p = 0.029), but there was no significant correlation for dead eggs (R^2^ = 0.07, p = 0.075). Also, the average egg portion covered by VECs was greater for both mature (0.4; p = 0.0025) and immature eggs (0.4; p = 0.0048) *vs*. dead eggs (Fig 3E). To understand the dynamics of encapsulation, co-cultures of mature or immature eggs and VECs were fixed at 0.5, 1, and 4 h, and imaged by confocal microscopy (S3 Fig). VECs migrated significantly faster (~3-fold) over both live immature and mature eggs compared to dead eggs (p < 0.0001 for both mature and immature eggs *vs*. dead eggs at 4 h; Figs 3F and S3). These data indicate that live eggs induce filopodia and NTs during encapsulation, whereas dead eggs provide less of a stimulus to the encapsulation process.

### Rho/ROCK-mediated VEC contractility is involved in the formation of filopodia and NTs, and the encapsulation of eggs

**Fig 4:**
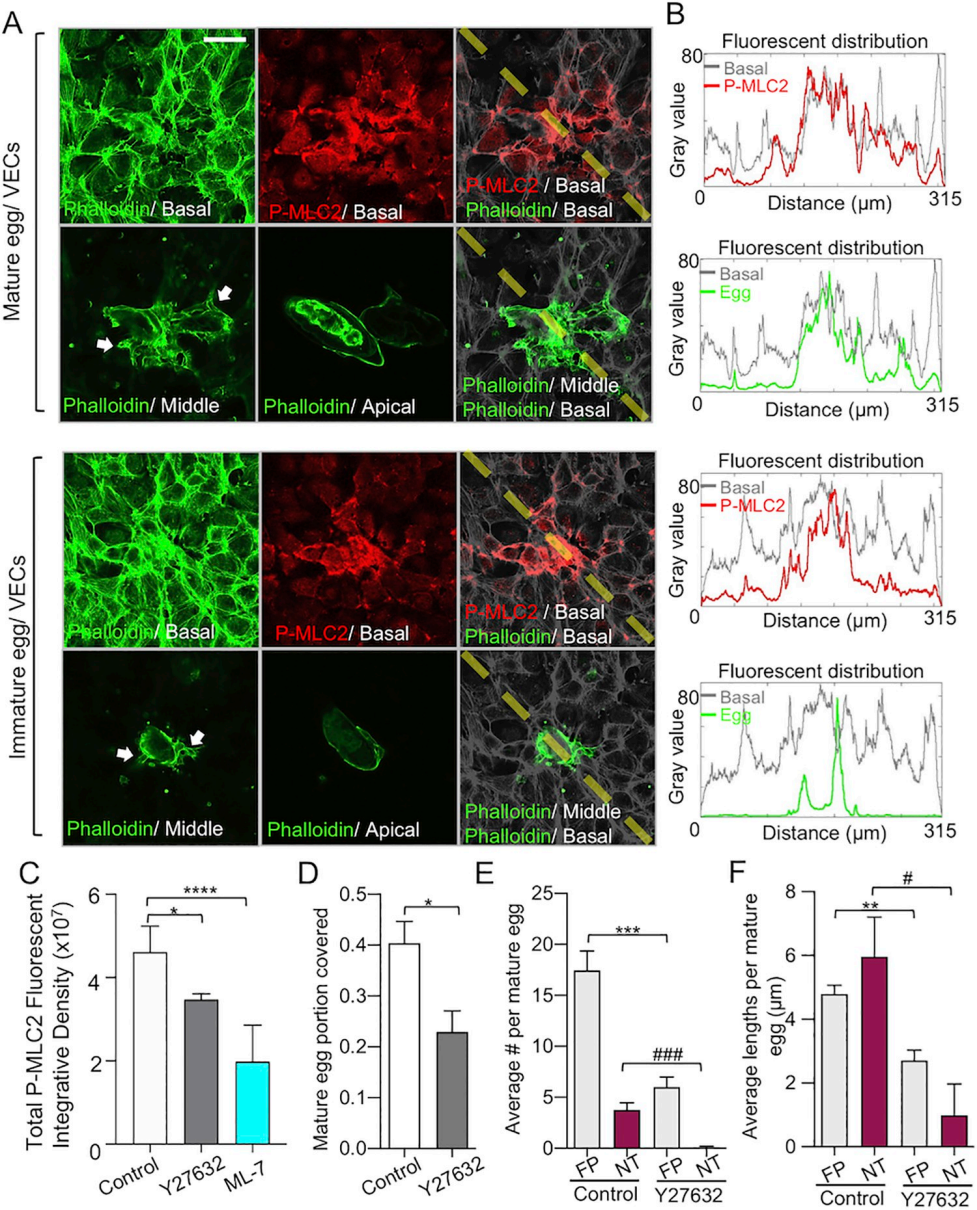
VEC contractility is involved in filopodia formation and encapsulation of eggs. **(A)** Immunostaining of phosphor-MLC2 and F-actin in VECs during encapsulation of live mature eggs at 4 h. The egg location is confirmed by different focal planes of VEC F-actin staining given that the eggshell is covered by VECs. The white arrows in the middle F-actin focal plane show the extended VECs surrounding the egg and indicate the corresponding egg location. **(B)** Fluorescent intensity analysis (the right panel) of phosphor-MLC2 basal (red line)/ F-actin basal (gray line) and F-actin middle (green line)/ F-actin basal (gray line) shows the distributions of phosphor-MLC2 and egg location across the merged images (yellow dashed line). Note how the induction of VEC myosin contractility localizes with the egg location (scale bar: 50 μm). **(C)** Phosphor-MLC2 fluorescent integrative density (mean ± s.e.m.) after 4 h of egg-VEC interaction. VECs were pre-treated with DMSO (white), Y27632 (Rho/RCOCK pathway inhibitor; gray) and ML-7 (myosin light chain kinase inhibitor; blue) for 30 min before incubating with eggs. * and ****, indicate p < 0.05 and p < 0.0001, respectively using a one-way ANOVA with Dunnett’s post-test. Six image fields from three independent experiments were analyzed. **(D)** Quantification of the mature egg portion covered by VECs (mean ± s.e.m.). * indicates p < 0.05 using Student’s *t*-test. **(E)** Quantification of the number (mean ± s.e.m.) of FPs and NT formed per mature egg. *** indicates p < 0.001 and ### indicates p < 0.001 using Student’s *t*-test. **(F)** Quantification of the average lengths of FPs and NTs (mean ± s.e.m.). ** indicates p < 0.01 and # indicates p<0.05 using Student’s *t*-test. For **D**–**F**, 16 and 11 eggs from three independent experiments were used for the control and Y-27632 treated VEC cases, respectively.

Having established that live eggs induce VEC filopodia and NTs during encapsulation, we investigated the mechanisms that may regulate VEC motility. Phosphorylation of the myosin regulatory light chain (MLC) is known to elicit the contraction of the actin cytoskeleton that facilitates cell motility [27]. Accordingly, we immunostained VECs for phosphorylation of MLC after a 4 h incubation with live mature and immature *S*. *mansoni* eggs. Confocal microscopy showed that active phosphor-MLC was more densely located within VECs in direct contact or closely associated with eggs in the basolateral focal plane of the VEC monolayers (Fig 4A and 4B). This pattern of increased phosphor-MLC in VECs was similar for both live mature and immature eggs. Pre-treating VECs with 20 μM Y-27632 or ML-7, small molecule inhibitors of Rho/ROCK and MLC kinase, respectively, significantly decreased the phosphor-MLC signal upon incubation with the live mature eggs (Fig 4C). The data demonstrate that live eggs induce VEC myosin activity and suggest that local myosin activity enables VEC contractility at egg contact sites to facilitate encapsulation.

Because the modulation of the cytoskeletal organization by MLC involved the Rho/ROCK pathway [27], we next pre-treated VECs with 20 μM Y-27632 for 30 min and quantified the ability of VECs to encapsulate live mature eggs after 4 h by SEM. Y-27632 decreased by 40% the portion of eggs covered by VECs compared to non-treated VECs (Fig 4D). Also, Y-27632 decreased the number of filopodia and NTs by 65 and 90%, respectively (Fig 4E), and the lengths of filopodia and NTs by 40 and 80%, respectively (Fig 4F). These data indicate the involvement of the Rho/ROCK signaling pathway in the VEC encapsulation process, including the formation of filopodia and NTs.

### Live eggs induce VECs to exert 3D forces to push eggs into the basement substrate

**Fig 5:**
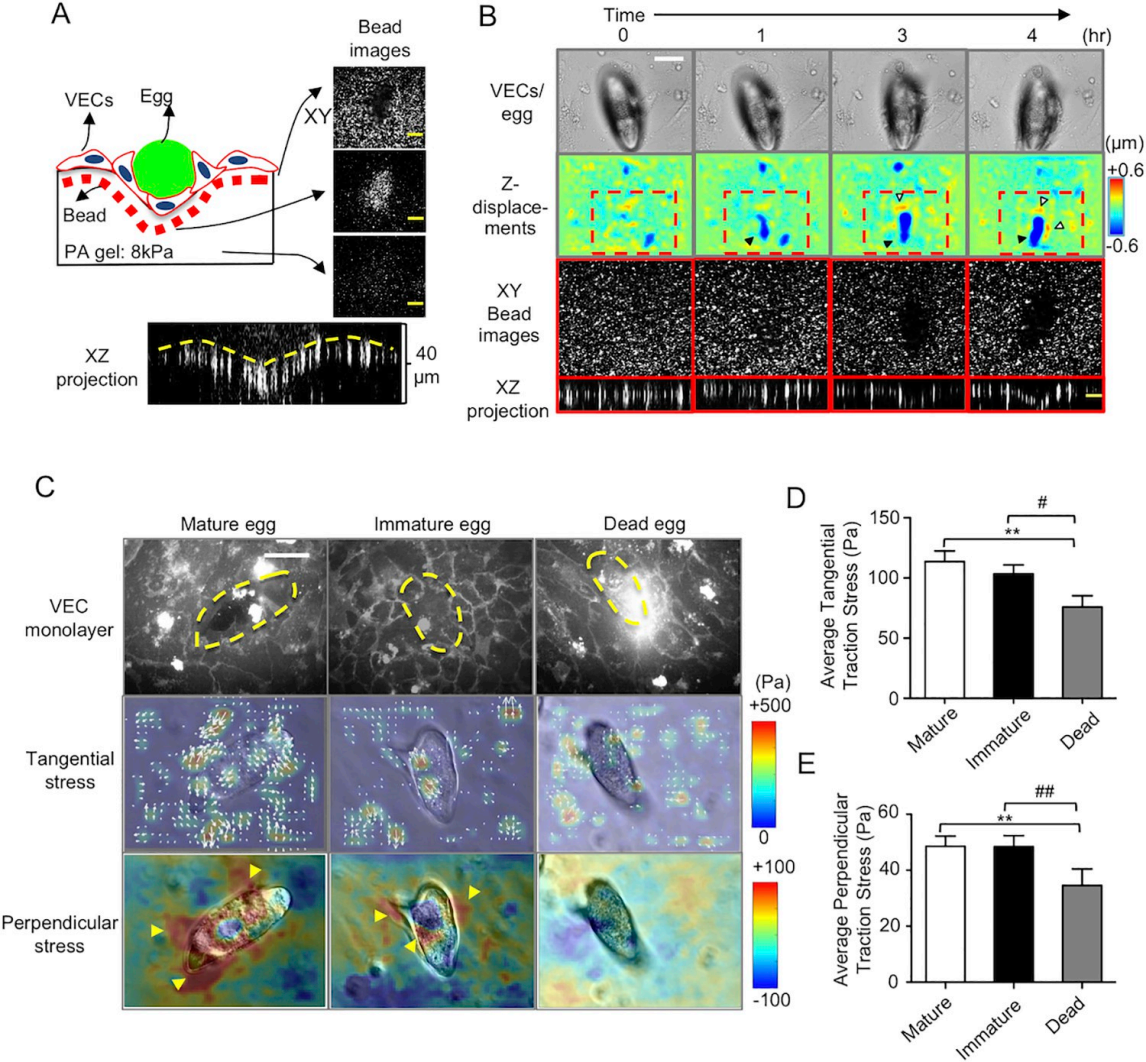
3D traction forces exerted by VECs during encapsulation of *S*. *mansoni* eggs. **(A)** Schematic of the 3D confocal microscopy setup to measure the 3D deformation of VECs during encapsulation. Fluorescent beads (red) were embedded in the elastic FN-coated elastic hydrogel substrates on which the VECs and eggs (green) were co-cultured. Orthogonal (xy and xz) views of the beads’ fluorescent channel demonstrate the 3D substrate deformation underneath the eggs (scale bar: 10 μm). **(B)** Temporal evolution of substrate deformation during encapsulation of a live mature egg. The top row shows bright-field images of the egg and VECs (scale bar: 50 μm). The second row shows maps of the z-displacement (perpendicular to the substrate) in the same field of view. Downward pushing patterns (negative z-displacement) are indicated by black arrowheads whereas upward pulling patterns (positive z-displacement) are indicated by white arrowheads. The third and fourth rows, respectively, show xy and xz enlarged images of beads in those areas with the greatest deformation (scale bar: 10 μm) as delimited by the red dashed-line box in the second row. **(C)** 3D traction stresses exerted by VECs interacting with live mature, live immature and dead eggs after 4 h. The top row shows VEC cell membrane staining (scale bar: 50 μm). The yellow dashed line shows the egg location. The second row shows the in-plane tangential (xy) stress magnitude (represented by the color map) and direction (white arrows). The third row shows the perpendicular (xz) stress magnitude. Positive/negative values indicate upward pulling and downward pushing, respectively. Note the downward pushing forces (blue color) localized at the egg anchoring site which is surrounded by upward pulling forces (red color, also indicated by yellow arrowheads). **(D-E)** Quantification of tangential **(D)** and perpendicular **(E)** traction stress magnitudes. Data are presented as the mean ± s.e.m. ** indicates p < 0.01, and # and ## indicate p < 0.05 and p < 0.01, respectively, using one-way ANOVA with Turkey’s post-test. For mature, immature and dead eggs, 26, 20 and 12 eggs were used, respectively, across three independent experiments.

To access the gut lumen and escape from the body, *S*. *mansoni* eggs must first cross the blood vessel’s basement membrane. The foregoing data regarding encapsulation suggest that mechanical forces may be generated by the VECs in contact with the egg. To investigate this process, we used elastic hydrogels (Young’s modulus E = 8 KPa) embedded with 0.2 μm fluorescent microbeads as substrates to culture VECs. These substrates mimic the mechanical characteristics of the native environment of the vessel tunica [28]. Measurement of the cell-generated traction forces is accomplished by embedded microspheres and then tracking their displacements. 3D traction force microscopy (3D-TFM) is used to calculate the 3-D traction forces from microsphere displacements [17,29,30]. After 24 h of interaction between a live mature egg and VECs, we imaged the displacement of the fluorescent microbeads in the hydrogel substrates at different focal planes (Fig 5A). The surface microbeads were pushed down to the middle focal plane as the egg volume deformed the subendothelial elastic hydrogel. The XZ projection showed the bending surface of the subendothelial layer over the 24 h interaction (Fig 5A).

Next, we performed a time-course experiment to investigate the dynamic changes of the VEC monolayer and subendothelial substrate in response to a live mature egg. After a 1 h interaction between egg and VEC, the substrate deformation had increased by over 2-fold in the z-direction perpendicular to the monolayer compared to the zero-time point (Fig 5B). After 3 and 4 h, the net z-deformations of the substrate by the egg had increased by up to 4-fold (S4 Fig and S3 Movie). Such downward deformations measured under the *S*. *mansoni* egg suggest that VECs actively generate 3D forces to push the egg into the tissue. To study whether egg vitality affects the force generated by VECs, we used 3D-TFM to quantify the forces exerted by VECs in contact with live mature eggs, immature eggs, and dead eggs for 4 h (Fig 5C). The 3D distributions of the traction force data showed that VEC tangential forces in the x-y direction had an irregular distribution of hotspots where the VECs were in direct contact with the egg (the second row of Fig 5C). Such force patterns have also been observed underneath endothelial monolayers when VECs interact with functionalized beads [17,31]. In contrast, the perpendicular forces in the z-direction had a more defined pattern with a downward pushing zone at the egg anchoring point and an upward pulling zone surrounding it (yellow arrowheads in the third row of Fig 5C). At 4 h, both the tangential and perpendicular forces were significantly stronger for mature and immature eggs relative to dead eggs, indicating that egg vitality is an important contributor to the process (Fig 5D and 5E).

### Increases in VEC monolayer tension during encapsulating cause VEC junctions to rupture

**Fig 6:**
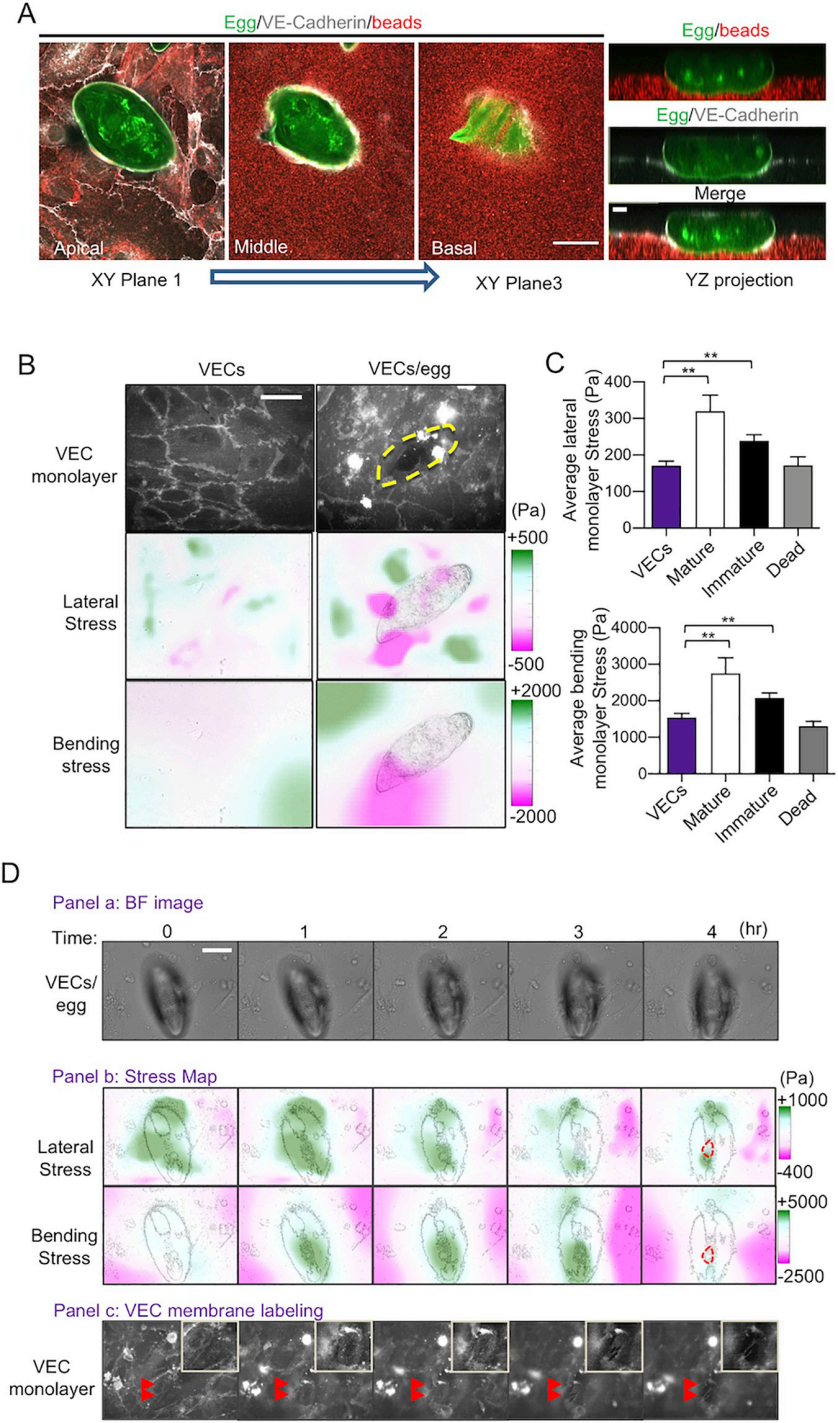
Increased monolayer tension during the encapsulation of *S*. *mansoni* ruptures VEC junctions. **(A)** Confocal images of a live mature egg being encapsulated by VECs after 4 h on a PA gel seeded with fluorescent beads. The three left-hand-side panels show xy images of VE-Cadherin staining (white), the egg (green) and beads within the gel (red), taken at the monolayer z-plane and then with increasing depth into the substrate (scale bar: 50 μm). The right-hand-side panel shows the corresponding yz projection (scale bar: 10 μm). **(B)** 3D stress patterns exerted by VEC monolayers in the presence and absence of a live mature egg after 4 h. The top row shows VEC junctions (scale bar: 50 μm). The yellow dash line outlines the egg location. The second and third rows represent lateral and bending monolayer tensions, respectively. **(C)** Quantification of the lateral (upper graph) and bending (lower graph) monolayer tension magnitudes (mean ± s.e.m.). Control VECs without eggs (n = 10), and VECs with 25, 20 and 12 live mature and immature, and dead eggs from three independent experiments, respectively, were analyzed. ** indicates p < 0.01 using Welch’s *t*-test. **(D)** Temporal evolution of the VEC monolayer during the encapsulation of a live mature egg. Panel a (bright field images): the first row shows images of the egg and VECs (scale bar: 50 μm). Panel c shows VEC junctions in the same field of view. Red arrowheads indicate an area where a junctional gap between the VECs has opened up underneath the egg (top right corner inserts show enlarged images of the region of interest). Panel b (stress maps): the first and second rows, respectively, show lateral and bending monolayer stresses superimposed on the egg’s silhouette. The red dashed contour at t = 4 h indicates the VEC gap location.

We examined whether the increased 3D deformations caused by the contact between VECs and parasite eggs resulted in endothelial barrier disruption. Specifically, we performed 3D confocal microscopy of immunostained VE-cadherin, the primary molecule regulating endothelial barrier function [32], during encapsulation (Fig 6A). VEC monolayers co-cultured with eggs for 4 h had disrupted VE-cadherin junctional connections co-localized with downward pushing sites and egg-hydrogel contacts. Moreover, VEC permeability was significantly increased in the presence of live mature eggs compared to either dead eggs or eggshells (S5 Fig).

To gain insight into the mechanism for endothelial disruption, we employed 3D monolayer stress microscopy (3D-MSM) [31] to quantify the changes in VEC monolayer tensions elicited by live *S*. *mansoni* eggs. The transmission of VEC contractile forces at cell-cell contacts raises the intracellular tension of the VEC monolayer by laterally stretching and bending the monolayer, and this tension can modulate the monolayer barrier function [33,34]. Our measurements revealed that VEC monolayers interacting with live mature and immature eggs generated greater lateral and bending tensions compared to monolayers without eggs (Fig 6B). Both the lateral (Fig 6C, upper panel; monolayer *vs*. mature, p = 0.0032; monolayer *vs*. immature, p = 0.0036) and bending components of the tension changed significantly (Fig 6C, lower panel; monolayer *vs*. mature, p = 0.0083; monolayer *vs*. immature, p = 0.0015). However, no statistical differences were measured between dead eggs and the monolayers alone.

Finally, we measured the monolayer tension over a 4 h period of VECs interacting with a mature egg, during which the VEC junctions underneath the egg became dissociated to eventually form a gap (Fig 6D panels a and c). Lateral tension (Fig 6D, panel b, 1st row) was initially strong (t = 0) and remained elevated for the first 2 h but decreased to more fluctuating patterns as the junctions began to dissociate at 3 h. On the other hand, the bending tension (Fig 6D, panel b, 2nd row) was initially weak (t = 0) but increased markedly over the next three hours as VECs encapsulated the egg and pushed it downwards. Eventually, the occurrence of a monolayer gap at the 4 h time point disrupted both the lateral and bending monolayer tension by creating a stress-free boundary along the gap perimeter. Such breaks in the integrity of the junctions were noted in approximately 70% of the live egg-VEC interactions observed. These data imply that the VEC monolayer actively alters its biomechanical state when in contact with live S. *mansoni* eggs and this, in turn, disrupts the integrity of VEC junctions and facilitates the passage of the egg. The time course of the data suggests that increased monolayer bending due to the egg being pushed into the basal membrane precedes loosening of the VEC junctions.

## Discussion

Migration through tissues by *S*. *mansoni* eggs is an essential process in the parasite’s life cycle. The necessary first step is contact between the parasite’s eggs and VECs which triggers intravascular host-immune responses to induce VEC inflammation, proliferation, and migration [6–8,35,36]. However, the biomechanical mechanisms regulating egg-VEC interactions are unknown. We used quantitative microscopy to show that S. *mansoni* eggs stimulate VECs to form membrane protrusions that facilitate the encapsulation of eggs. When VECs came into contact with eggs, they exerted 3D mechanical forces mediated by the activation of Rho/ROCK signaling that pushed the egg towards the basement membrane. The increased VEC contractility also caused monolayer tension to nearly double and fluctuate, thus, destabilizing cell-cell junctions and facilitating egg extravasation. Importantly, we demonstrate that the mechanisms described are enhanced by egg vitality, whereby dead eggs are far less stimulatory. Overall, our data describe a mechanism by which *S*. *mansoni* eggs hijack the contractile machinery of the host’s endothelium to propel their motion toward the extravascular space.

The eggshell is a tanned and rigid structure composed of various cross-linked proteins and has a dense distribution of surface projections, commonly referred to as microspines (see [23,37–41] and references therein). We studied the interactions between the egg microspines and VEC membrane protrusions at the nanometer scale using SEM. Filopodia are thin, actin-rich bundled fibers that protrude from cell membranes and serve several physiological roles, such as probing the environment and facilitating cell motility [42]. Apart from filopodia, recent studies have identified another type of cell membrane protrusion, named intercellular NTs or tunneling NTs, which do not attach to the substrate but establish connections between cells and are thought to mediate long-range intercellular communication [25]. These NTs can transfer cytoplasmic material and pathogens such as HIV from one cell to another [26,43]. In VECs, the formation of intercellular NTs can be stimulated by oxidative stress, apoptosis, and inflammation [44,45]. Interestingly, we observed that VECs generated both filopodia and NTs when they were in direct contact with S. *mansoni* eggs. Filopodia appeared at the front of the “leader” VECs to probe the egg surface, whereas intercellular NTs were found at the trailing edge of these leader cells to connect with the basal endothelium. Filopodia extended from the VEC cytoskeleton and firmly adhered to the eggshell, and the number of these thin frontal protrusions positively correlated with the eggshell area covered by the VECs.

Collective cell migration relies on the long-range coordination of cell polarity between leader and follower cells [46]. Our data suggest that filopodia and NTs regulate collective cell polarity during egg encapsulation. We determined that the leading filopodia on VECs exhibited a ‘seeking’ or ‘exploration’ phenotype, advancing onto a new egg before completely covering those that had already been engaged (Fig 2B). Adherens junctions have been implicated as major contributors to collective polarity by transmitting cellular forces across flat monolayers [15]. In contrast to adherens junctions, NTs mediate long-range collective communication over distances of up to 100 μm in leukocytes [47]. We observed endothelial cell NTs as long as 20 μm in our experiments mediating long-range cell-cell connections during the migration of VECs over eggs. Thus, we propose that NTs could reinforce the collective VEC behavior during encapsulation in a manner similar to their functionality in the embryonic cell sheet-like migration model during which leader cells at the migratory front extend intercellular NT bridges to help pull the cells behind [48].

The present data indicate that live eggs increase VEC migratory activity. VECs encapsulated both mature and immature live eggs at similar rates, covering ~40% of the eggshell surface within the first 4 h of interaction, whereas the encapsulation of dead eggs was significantly less prominent (~20%) over the same period. Furthermore, VECs interacting with living eggs for 4 h had ~2.5- and 10-fold more filopodia and intercellular NTs than those interacting with dead eggs over the same period. It is known that immature eggs released by the female worm onto the mesenteric veins induce rapid VEC encapsulation without inducing granuloma formation due to eggshell proteins and egg-bound platelets, Von Willebrand factor and plasma proteins, possibly aided by worm-endothelium interactions [6,13,39,49–51] and eggshell surface topography (S3 Fig). Also, ESPs from mature eggs activate VEC adhesion, migration and proliferation, in addition to promoting angiogenesis and recruitment of leukocytes to initiate the granulomatous response, thereby facilitating egg migration [6–8,52–57]. The difference in migratory activity between living and dead eggs measured here might be due to ESPs from the developing embryo [55,58–60] released via the microscopic eggshell pores [61,62]. Whereas previous studies showed that the *attachment* by *S*. *mansoni* eggs to VECs was not affected by whether parasite eggs were alive or killed using glutaraldehyde [53], our study quantified the *encapsulation* dynamics of eggs by VECs and, consistent with previous findings which employed formalin-fixed eggs [6], the data show an initial delay in the migration of VECs over dead eggs compared to live mature and immature eggs (Fig 3F).

In addition to vitality, the egg’s developmental state influenced the dynamics of the formation of filopodia and NTs. Thus, the filopodia on dead and live mature eggs had similar lengths (~4–5 μm), whereas those on live immature eggs were twice as long. We also found that VECs migrated slightly faster over live immature eggs compared to mature eggs (13–18 μm *vs*. 8–13 μm in the z-direction between 0.5 and 1 h). We speculate that the longer VEC filopodia on immature eggs could contribute to faster VEC encapsulation possibly mediated by the mechanisms referred to above. Although our data indicate the importance of vitality and maturation state of the eggs as harvested from the liver, immature eggs freshly deposited by female worms might generate additional relevant information. However, difficulties associated with reliably generating sufficient eggs from female worms *in vitro* would have made the performance of the experiments described challenging. We also note that the remodeling induced by schistosome eggs described here for VECs may be cell-type specific. For example, in the case of perivascular fibroblasts, mature egg ESPs kill these cells to facilitate their migration through tissues [63]. Also, previous work showed that *S*. *mansoni* eggs attach to HUVEC and bovine aortic endothelial cell monolayers faster than to fibroblasts and smooth muscle cell monolayers [53].

During cell migration, actomyosin-based contractility integrates the filopodial F-actin bundles into protruding lamellipodia at the cell front [42]. The Rho/ROCK pathway is a key regulator of cell migration that coordinates cell polarity and contractility [27]. Specifically, triggering of the Rho/ROCK pathway activates myosin II, which mediates cytoskeletal remodeling and modulates intracellular tension to control the dynamics of cell adhesion [64]. In this context, our experiments showed that MLC phosphorylation was enhanced in VECs in direct contact with *S*. *mansoni* eggs, implying that myosin II contractility had been activated. Moreover, small molecule inhibition of the Rho kinase activity in VECs decreased their ability to encapsulate eggs, as well as the length and number of filopodia, and intercellular NTs. These results highlight the importance of the VEC Rho/ROCK pathway in driving VEC motility during the early stages of egg extravasation. Furthermore, our data suggest that *S*. *mansoni* eggs modulate the contractile machinery of the surrounding vascular cells, which motivated us to investigate whether the resulting forces generated by the VECs are sufficient to physically drive the eggs across the endothelium.

S. *mansoni* eggs cannot move independently and must rely on external forces to transit from the vasculature toward the intestinal lumen. Given the egg’s voluminous size and rigidity (dimensions ~100 x 30 x 30 μm and Young’s modulus ~10 MPa), this extravasation process likely causes large deformations in the egg’s surroundings and requires significant forces. The origin of these forces has been the subject of speculation in the past, leading to two main theories. One theory suggests that the eggs recruit endothelial cells to push them into the basal membrane and promote proteolysis of the extracellular matrix [6,12]. An alternate theory proposes that muscular contractions of the female worm force the eggs into the vessel wall [13]. To shed light on these questions concerning the eggs, we used 3D-TFM to measure the external forces involved during the interaction of VECs with eggs *in vitro*. Using soft, elastic hydrogels embedded with 0.2 μm fluorescent microspheres as cell substrates, we mimicked the mechanical properties of the egg’s physiological environment [65]. Significant substrate indentations and bending of the VEC monolayer occurred under the eggs during the first hour of encapsulation, and these increased in magnitude over time. The tangential (in-plane) traction stresses exerted by the VECs increased ~1.6-fold under mature and immature eggs compared to dead eggs, showing a disordered vector pattern. In addition, perpendicular stresses also increased ~1.6-fold and appeared under the eggs, displaying a peripheral ring of upward pulling traction surrounding a small area of intense downward pushing (S6 Fig). This pattern of 3D traction stress is consistent with that observed at cell-cell junctions during the engulfment by VECs of inert particles coated with ICAM [17]. Given that VEC ICAM expression is strongly upregulated upon exposure to egg ESPs [54], we posit that eggs activate VECs to generate forces strong enough to push the eggs across the endothelium. Finally, the large lateral spine of the S. *mansoni* egg has been speculated to play a role in egg extravasation [66]. Accordingly, we analyzed the substrate indentation sites and the egg spine positions (S7 Fig) and found that they did not co-localize. Thus, it seems that the egg spine does not contribute to the initial stages of egg extravasation.

The ability of the *S*. *mansoni* egg to promote 3D forces from VECs depends, to a quantitative degree, on the vitality of the *S*. *mansoni* egg. Specifically, we found that VECs in contact with live eggs generated traction stresses that were at least twice as strong as those generated by VECs in contact with dead eggs. The VEC monolayers in contact with live eggs also had a larger intracellular tension than those in contact with dead eggs. Under increased tension, VEC monolayers have loosened adherens junctions and increased migration, leading to transient endothelial gaps [16,17]. Indeed, we found that live eggs often caused the breakdown of VEC junctions with an increase in endothelial permeability. Thus, we postulate that the modulation of VEC contractility and monolayer tension are orchestrated by secretions from the live egg, which not only force the egg into the vessel wall but also shift the VEC monolayer to a state of decreased barrier function. Together, our findings demonstrate how the biomechanical interactions between *S*. *mansoni* eggs and VECs are critical for successful egg extravasation.

Successful passage of the *S*. *mansoni* egg from the endovascular space to the lumen of the gut is essential for continuation of the parasite’s lifecycle. Although it is well-established that schistosome eggs are biologically active and release factors that modulate the immune response during tissue migration (cited above), the biomechanical processes at play in the first and crucial steps of egg encapsulation and extravasation, have been less studied. Our investigations comparing live mature and immature, and dead *S*. *mansoni* eggs, have discovered how egg vitality contributes to activating VECs to encapsulate and exert 3D mechanical forces on eggs that eventually disrupt VEC junctions to transport eggs away from the endothelial surface into the underlying tissues. Future work will examine specific components of the eggs for their possible contributions to the biomechanical processes described and measured here, including possible differences between schistosome species.

## Materials and methods

### Ethics statement

Male Golden Syrian hamsters infected with the Naval Medical Research Institute (NMRI, Puerto Rican) isolate of *S*. *mansoni* were obtained from the Biomedical Research Institute (BRI, Rockville, MD) [67] and maintained in accordance with protocols approved by the Institutional Animal Care and Use Committee (IACUC) at the University of California San Diego.

### Reagents

The VE-Cadherin antibody used for immunostaining was purchased from Santa Cruz Biotechnologies (sc-9989). The antibody against phosphor-MLC2 serine 19 was purchased from Cell Signaling Technology (3671). Alexa Fluor-488-, Alexa Fluor-594-Phalloidin and Cell Mask deep red plasma membrane stain used for immunostaining and/or live cell labeling were purchased from Invitrogen (A12379, A12381 and c10046). The carboxylate-modified red (580/605) microspheres used for traction force microscopy were purchased from Invitrogen (F8809). Y27632 was purchased from Abcam (ab120129) and ML-7 was purchased from Sigma (475880).

### Isolation of *S*. *mansoni* eggs

Eggs were isolated from the livers of hamsters six weeks after infection with 600 *S*. *mansoni* cercariae [68]. Specifically, livers were finely minced with a sterile razor blade and digested overnight at 37°C in 40 mL 1×PBS containing 2% penicillin/streptomycin/amphotericin B (1×PBS -2% PSF) and 5 mL 0.5% clostridial collagenase solution (0.025 g of collagenase (Sigma, C0130) in 5 mL of dH_2_0 for immediate use). All subsequent steps proceeded at room temperature. The digested livers were centrifuged at 400 × *g* for 5 min and the supernatant was decanted. The resulting pellet was resuspended in 50 mL 1×PBS-2% PSF, centrifuged under the same conditions and the supernatant decanted: this step was repeated 3–5 times until the supernatant was clear. During this process, eggs settle to the bottom of the conical tube. Eggs were layered gently onto the first Percoll gradient (8 mL sterile Percoll with 32 mL 0.25 M sucrose) and centrifuged at 800 × *g* for 10 min to separate any remaining liver tissue debris. The supernatant was discarded with a serological pipette. Eggs were resuspended in 3 mL 1×PBS -2% PSF and gently applied to the second Percoll gradient (2.5 mL Percoll with 7.5 mL 0.25 M sucrose). Immature eggs were removed at the interface and mature eggs were collected from the bottom of the column. Immature eggs were further separated using a third Percoll gradient (6 mL of Percoll, 0.6 mL 9% saline and 3.4 mL M199 culture medium) and centrifugation for 15 min at 250 x *g* [55]. The mature and immature egg fractions were washed 3–5 times with M199 medium, centrifuged at 310 x *g* for 3 min to remove any remaining Percoll and tissue debris. Mature egg fractions typically contain less than 5% immature eggs, whereas immature egg fractions contain 5–10% mature eggs, as observed microscopically. Hatching of mature eggs in water was usually ~80% after 40 min under a bright light, thus confirming their viability. Egg size and nuclear status were checked to validate the egg developmental stage. Immature eggs were smaller, and under bright field images, there were no clear envelopes separating the embryo from the eggshell, indicating an undeveloped status [22]. In contrast, mature eggs were larger with a well-defined envelope containing a moving miracidium (S1 Table and S2A Fig). To generate dead eggs, mature eggs were treated with 1% sodium azide (Sigma S2002) for 24 h in M199 and then washed six times in the same medium. All experiments were conducted within five days of preparing eggs.

### Cells and co-culture

Human umbilical VECs (Cell Application 200-05n) were cultured on fibronectin (Sigma 10838039001)-coated glass slides or polyacrylamide (PA) hydrogels for 48 h in M199 supplemented with 10% endothelial cell growth medium (Cell Application), 10% FBS (Lonza), 1% sodium pyruvate, 1% L-glutamine and 1% penicillin-streptomycin (Gibco) until they formed a confluent monolayer. Eggs were added on top of the VECs at a density of 4 eggs /mm^2^ (~500 eggs per 12 mm diameter round coverslip) and co-incubated for up to 24 h.

### Immunofluorescence and confocal microscopy

Cells were cultured on fibronectin-coated substrates (50 μg/mL) and treated with eggs or reagents at the indicated time points. After treatment, cells were fixed in 4% paraformaldehyde for 10 min. Cells were then permeabilized in 1×PBS supplemented with 0.1% Triton X-100 for 10 min followed by a blocking step in 1×PBS supplemented with 5% BSA (Sigma A7906). Cells were incubated with primary and secondary antibodies and, after each step, washed with 1×PBS. DAPI (Sigma 10236276001, 1μg/mL) was used as a counterstain. Images were taken with an epi-fluorescence microscope (Olympus IX70) or a confocal microscope (Zeiss 880 Airyscan). For 3D-TFM, z-stack image acquisition was performed using a spinning disc confocal microscope (Olympus IX81), a 40X NA 1.35 oil immersion objective lens, a cooled CCD (Hamamatsu) camera and the Metamorph software version 7.8.10 (Molecular Devices). For the 3D image analysis, sequences of taken z-stack images were taken and analyzed using the Volocity software version 6.3 (Quorum Technologies Inc.) which rendered the optical sections as 3D models, thus enabling the analysis of the interactions between parasite eggs and VECs.

### Scanning Electron Microscopy (SEM)

Samples were fixed with 2% glutaraldehyde (Electron Microscopy Sciences) and 4% paraformaldehyde, pH 7.4, for 24 h. Samples were critical point dried in increasing concentrations of high-grade ethanol using an Autosamdri 815 critical point dryer and then sputter coated with iridium using an Emitech K575X. Imaging was done with a QUANTA FEG 250 ESEM (Field Electron and Ion Company) or Sigma 500 SEM (Zeiss). For each image, the average length and number of filopodia and intercellular NTs was measured and counted using the FIJI imaging analysis software [69].

### Polyacrylamide (PA) gel preparation and characterization

On 35-mm glass-bottom dishes (World Precision Instrument FD35-100), we fabricated 12 mm diameter and 40 μm thick PA gels in 1×PBS by first mixing 5% acrylamide and 0.3% bis-acrylamide (Young’s modulus E = 8.7 KPa) and then adding a 1/100 total volume of 10% APS and a 1/1000 total volume of TEMED (Sigma; to initiate gel polymerization). To improve the signal to noise ratio of the z-stack images and the displacement field calculation, the PBS used in the fabrication of the gels contained 0.03% carboxylate-modified red microspheres (0.2 μm diameter; Thermo Fisher Scientific). After placing a coverslip on top, the side was inverted and the gel allowed to polymerize for 30 min, during which time the microbeads migrated to the surface of the gel. The distribution of the microbeads at the surface of the gels was verified by the 3D confocal microscopy. To encourage cell attachment to the PA gels, we crosslinked the extracellular matrix protein, fibronectin, to the gel surface by using UV activated Sulfo-SANPAH (0.15 mg/mL; Thermo Scientific 22589). The gels were incubated overnight at 4°C.

### Three-Dimensional Traction Force Microscopy (3D-TFM) and Monolayer Stress Microscopy (MSM)

The 3D deformation of the PA gel’s surface, in which the fluorescent microspheres were localized, was measured with a confocal microscope (Olympus IX81). The 3D deformation field was formulated as ***u***(*x,y,z* = *h*), where the deformation vector ***u*** depends on the coordinates of *x,y,z* and for a single horizontal plane measurement, *z = h*. Time-lapse sequences of fluorescence z-stacks consisting of 40 planes separated by 0.4 μm were acquired at 1 h intervals. The 3D deformation was determined by cross-correlating each instantaneous z-stack with an undeformed reference z-stack taken after detaching the cells by trypsin treatment. After imaging, attached cells were trypsinized and their removal relaxed the elastic substrates back to the undeformed state that served as reference for the correlations. To balance the spatial resolution and signal-to-noise ratio, the *z-*stacks were divided into 3D interrogation boxes of 32 × 32 × 12 pixels in the *x*, *y*, and *z* directions, respectively. These settings provided a Nyquist spatial resolution of 2 μm in three spatial directions. The elasticity equation of equilibrium was solved for a linear, homogeneous and isotropic body with a Poisson’s ratio σ = 0.45 [29,30,70] to determine the 3D deformation everywhere inside the substrate, ***u***(*x,y,z*), from ***u***(*x,y,h*). We then applied Hooke’s law to calculate the six independent components of the stress tensor everywhere inside the substrate. In particular, we computed the 3D traction stress vector at the surface of the substrate in contact with the cells, $\left[\tau_{xz}(x,y,h),\;\tau_{yz}(x,y,h),\;\tau_{zz}(x,y,h)\right]$. We used 3D MSM to infer the intracellular tension caused by lateral deformation and bending of cell monolayers [31]. The calculation is carried out by imposing equilibrium of forces and moments in the monolayer subject to external loads given by the 3D traction stresses.

### Statistical analysis

For comparisons between two groups, statistical analyses were performed by two-tailed unpaired Student’s *t-*test and Welch’s *t*-test. Comparison of multiple groups was made by one-way ANOVA, and statistical significance among multiple groups was determined by Tukey’s post-test or Dunnett’s post-test (for pair-wise comparisons of means). All results are presented as means ± s.e.m. from three independent experiments.

## Supporting information

S1 Table Mature and immature *S*. *mansoni* egg properties. To measure the long and short axis, and height, 11 each of mature and immature eggs were used. For each parameter, the comparison between mature and immature eggs was significant: ***, p<0.001 by Student’s *t*-test. To measure volume, seven eggs were used, and the comparison between mature and immature eggs was significant: * p<0.05 by Student’s *t*-test. To measure egg stiffness, two eggs in each case were probed by Atomic Force Microscopy.
(TIF)

S1 Fig Morphology, hatching ability and staining of mature and immature *S*. *mansoni* egg embryos. **(A)** Mature and immature eggs were cultured for 24 h and then hatching attempted in distilled water under a bright light for 40 min. Unlike mature eggs, of which ~80% had hatched (empty eggshells indicated by arrowheads), immature eggs cannot hatch. Scale bar, 200 μm. All eggs in the experiments were not hatched. **(B)** Images of mature and immature eggs: DAPI was used to stain egg embryo nuclei. Scale bar, 20 μm.
(TIF)

S2 Fig VEC filopodia contain F-actin filaments. **(A)** The *S*. *mansoni* egg was encapsulated by VECs at 4 h. (a) Z-stack images (See SI movie 1) of the distribution of F-actin from the apical to the basolateral focal plane. (b) The basolateral image of the stack images. Scale bar = 50 μm. **(B)** Area of interest, indicated by the white dashed line box in (**A**), enlarged with its component Z-stack sections displaying the stained VEC filopodia.
(TIF)

S3 Fig The kinetics of VEC encapsulation of *S*. *mansoni* eggs. Representative 3D images of the interaction between mature and immature eggs during the VEC encapsulation process. VECs were immunostained by Phalloidin (red), eggs were auto-fluorescently green, and cell and egg nuclei were immunostained by DAPI. Extended focus images combine the in-focus parts of each image together to produce a single image with an increased depth of field. Scale bar = 50 μm. Z-direction images show the progress of VEC coverage of eggs. The total height is 60 μm. White arrowheads indicate VECs migrating on the side and over the eggs.
(TIF)

S4 Fig Quantification of peak deformation and gel deformed volume during encapsulation of a live mature *S*. *mansoni* egg. To obtain the quantification result for **Fig 5B**, a cropped area based on peak z-direction displacements was selected at each time point as shown in the red dashed box (**Fig 5B**) to fit to a 2D Gaussian curve. The peak deformation and deformed gel volume obtained in the z-direction are in absolute values.
(TIF)

S5 Fig VEC permeability as a function of *S*. *mansoni* egg vitality. **(A)** Live and dead (treated with sodium azide) mature eggs, and eggshells only were incubated with VEC monolayers for 24 h. Apical bright field images were taken. VE-Cadherin (red) and DAPI (blue) were immunostained in the VECs present on the eggshell surface and show how the cells can encapsulate the three egg preparations after 24 h. Scale bar, 50 μm. **(B)** Permeability response of VEC junctions after interaction with live or dead eggs, or eggshells for 4 h. The control condition is VECs monolayers without eggs. Data represent the mean ± s.e.m. **P<0.01, # P<0.05 and ## P<0.01 using the one-way ANOVA with Tukey’s multiple comparison test. Three independent experiments were performed.
(TIF)

S6 Fig Schematic of the tangential and perpendicular stresses during encapsulation of a *S*. *mansoni* egg. Endothelial cells encapsulating a *S*. *mansoni* egg (green oval) collectively generate pulling upward and inward forces (indicated by red arrows) onto the subendothelial substrate (e.g., basement membrane). These forces are exerted as the leading edge of the endothelium climbs up and over the egg and are required to balance the downward pushing forces that endothelial cells exert on the egg. The downward pushing forces are transmitted to the substrate under the egg’s central region (purple arrows). In comparison, the horizontal forces generated by contraction of individual cells contribute a more disorganized tangential stress pattern (black arrows). Due to the downward forces exerted by the endothelial cells that overlie the egg, the integrity of the endothelial monolayer directly underneath the egg is compromised, eventually breaking the cell-cell junctions. *In vivo*, this process would force the egg into the underlying tunica layers of the blood vessel, thus initiating the migration of the egg away from the blood vessel.
(TIF)

S7 Fig Analysis of the spatial localization of the *S*. *mansoni* egg’s lateral spine and the anchoring point relative to the egg basal centroid. **(A)** As introduced in Fig 5A and 5B, we defined the center of the anchoring point by fitting the ellipse shape for the z-deformation region (a). In addition, we define the egg center by fitting the ellipse shape for the egg shape (b). Scale bar, 50 μm. **(B)** The distance from the egg center to the egg’s lateral spine (green line) and from the egg center to the anchoring point (pink) were calculated by using the FIJI imaging processing software. The major axis of the egg was used to normalize for the variation in the size of each egg. **(C)** Quantification of the distance from the egg’s center to the lateral spine and to the anchoring point. Data were normalized to the length of the long axis of each egg. For each condition, 12–15 eggs were used: ****, p<0.0001 by Student’s *t-*test.
(TIF)

S1 Movie Z-stack images of F-actin staining show that VEC filopodia exert on the *S*. *mansoni* egg surface. (AVI)

S2 Movie Time-lapse images show the movement of a live *S*. *mansoni* miracidium during the encapsulation of mature eggs by VEC after 24 h (corresponding to Fig 5A). (AVI)

S3 Movie Time-lapse images show the interaction between VECs and a live mature *S*. *mansoni* egg, and the 3-D deformation on the soft substrates during encapsulation (corresponding to Fig 5B). (AVI)

S1 Materials and Methods Dextran permeability assay. Confluent VECs were cultured in fibronectin-treated 6-well cell culture inserts (Corning, 0.4-μm pore) for 48 h. Five hundred mature eggs, eggshells or dead eggs were added into the transwell insert. Immediately after, FITC–dextran (100 μg; 40 KDa; Sigma) in HEPES buffer was added to the upper chamber. Every 30 min for up to 4 h, we collected 50 μL samples from the lower chamber each time replacing the volume in the upper chamber with M199 medium to maintain hydrostatic equilibrium. Samples were diluted to 1 ml with PBS and 100 μl transferred to 96-well black plates (ThermoFisher) to measure the fluorescence content at 492/520 nm absorption/emission wavelengths in a TECAN fluorometer.
(DOCX)
