# Supplementary material for: Biomechanical interactions of Schistosoma mansoni eggs with vascular endothelial cells facilitate egg extravasation
Source: PLoS Pathog. 2022 Mar 22;18(3):e1010309. doi: 10.1371/journal.ppat.1010309 (PMC8939816; doi:10.1371/journal.ppat.1010309)
Supplement: S1 Materials and Methods — Confluent VECs were cultured in fibronectin-treated 6-well cell culture inserts (Corning, 0.4-μm pore) for 48 h. Five hundred mature eggs, eggshells or dead eggs were added into the transwell insert. Immediately after, FITC–dextran (100 μg; 40 KDa; Sigma) in HEPES buffer was added to the upper chamber. Every 30 min for up to 4 h, we collected 50 μL samples from the lower chamber each time replacing the volume in the upper chamber with M199 medium to maintain hydrostatic equilibrium. Samples were diluted to 1 ml with PBS and 100 μl transferred to 96-well black plates (ThermoFisher) to measure the fluorescence content at 492/520 nm absorption/emission wavelengths in a TECAN fluorometer. (DOCX) [file ppat.1010309.s012.docx]

**Supplementary Materials and Methods
Dextran permeability assay**

Confluent VECs were cultured in fibronectin-treated 6-well cell culture inserts (Corning, 0.4-μm pore) for 48 h. Five hundred mature eggs, eggshells or dead eggs were added into the transwell insert. Immediately after, FITC–dextran (100 μg; 40 KDa; Sigma) in HEPES buffer was added to the upper chamber. Every 30 min for up to 4 h, we collected 50 μL samples from the lower chamber each time replacing the volume in the upper chamber with M199 medium to maintain hydrostatic equilibrium. Samples were diluted to 1 ml with PBS and 100 μl transferred to 96-well black plates (ThermoFisher) to measure the fluorescence content at 492/520 nm absorption/emission wavelengths in a TECAN fluorometer.
